# Non-gravitational acceleration indicative of cometary activity of near-Earth object

**DOI:** 10.1038/s41550-026-02913-7

**Published:** 2026-07-10

**Authors:** D. Farnocchia, O. R. Hainaut, D. Z. Seligman, R. Weryk, L. A. M. Benner, M. Brozović, L. Denneau, A. Galád, J. D. Giorgini, K. Hornoch, M. Micheli, S. P. Naidu, R. S. Park, P. Pravec, R. J. Wainscoat

**Affiliations:** 1 Jet Propulsion Laboratory, California Institute of Technology, Pasadena, CA USA; 2 European Southern Observatory, Garching bei München, Germany; 3 Department of Physics and Astronomy, Michigan State University, East Lansing, MI USA; 4 Physics and Astronomy, The University of Western Ontario, London, Ontario Canada; 5 Institute for Astronomy, University of Hawaii, Honolulu, HI USA; 6 Astronomical Institute AS CR, Ondřejov, Czech Republic; 7 ESA NEO Coordination Centre, Planetary Defence Office, Frascati, Italy

**Keywords:** Asteroids, comets and Kuiper belt, Early solar system

## Abstract

The activity of comets when they approach the Sun usually manifests visibly with the appearance of a dust and gas coma, and in turn non-gravitational accelerations perturbing their motion. However, there exist objects that are morphologically inactive and yet exhibit non-gravitational accelerations. Here we show that astrometry from 1998 to 2025 of near-Earth object (875163) 1998 SH_2_ reveals orbital perturbations consistent with cometary outgassing. Although no cometary activity was evident in archival images, large-aperture telescopic observations show a weak low-surface-brightness tail and prove that 1998 SH_2_ is indeed a cometary object. When cometary activity is weak, outgassing may remain undetected for decades. Our results show that non-gravitational perturbations apparent over long astrometric data arcs provide a diagnostic for identifying comets and suggest that more near-Earth objects currently classified as asteroids could in fact be comets. Therefore, our findings prompt searches to detect additional near-Earth objects exhibiting weak cometary activity. The motion of these weak comets can be more strongly perturbed than that of asteroids, influencing assessment of their Earth impact hazard and our understanding of the delivery of water to Earth.

## Abstract (web summary)

Subtle orbital shifts prompted deep imaging of near-Earth object 1998 SH_2_, which uncovered a weak tail and confirmed cometary activity. The result indicates that more near-Earth objects currently classified as asteroids may also exhibit hidden low-level activity.

## Main

Asteroids and comets are classically differentiated based on their orbits, composition, and observed cometary tails and activity levels^1^. However, in recent years, a continuum of hybrid objects have become more apparent^2^, eroding the traditional boundaries between these two classes. For example, some asteroids have been found to display comet-like activity and are referred to as active asteroids^3,4^. Conversely, there are distinct populations of objects such as ‘Manx comets’^5^ and Damocloids^6–8^ that are on comet-like orbits but do not display cometary activity—at least during extant serendipitous surveys and targeted observations. This continuum of activity spanning small-body populations is not unique to our Solar System. Unlike 2I/Borisov^9^ and 3I/ATLAS^10^, the first discovered interstellar object, 1I/‘Oumuamua, showed no evidence of a cometary dust tail in deep imaging^11–13^. However, 1I/‘Oumuamua was clearly affected by non-gravitational accelerations, suggesting the possibility of undetected cometary outgassing^14^.

In recent years, 14 near-Earth objects have been identified as inactive yet subject to non-gravitational perturbations^15^, seemingly inconsistent with radiation forces that perturb the motion of asteroids^16–18^. The appropriate classification of these enigmatic objects as asteroids or comets remains unclear; they are informally referred to as ‘dark comets’. These objects have been divided into two distinct populations: inner dark comets, which are tens of metres in size or smaller and have orbits close to that of the Earth, and outer dark comets, which are hundreds of metres in size or larger and have orbits similar to those of Jupiter family comets. For the inner dark comets, non-gravitational perturbations exhibit a term in the direction perpendicular to the orbit plane, whereas radiation forces are mostly in the orbital plane^19,20^. Recent refinements to radiation models that take into account asymmetries in the shape of the asteroid may explain the direction of the observed non-gravitational forces on inner dark comets^21^. For the outer dark comets, the magnitude of the detected non-gravitational perturbations is simply too large to be caused by radiation effects and may instead result from outgassing, with cometary activity below the sensitivity threshold of telescopic observations. For one of these objects, (139359) 2001 ME_1_, this interpretation was confirmed by the recent detection of activity in images from the Solar and Heliospheric Observatory taken in 2018, when the object was near perihelion^22^.

## Non-gravitational perturbations on the motion of 1998 SH_2_

**Fig. 1:**
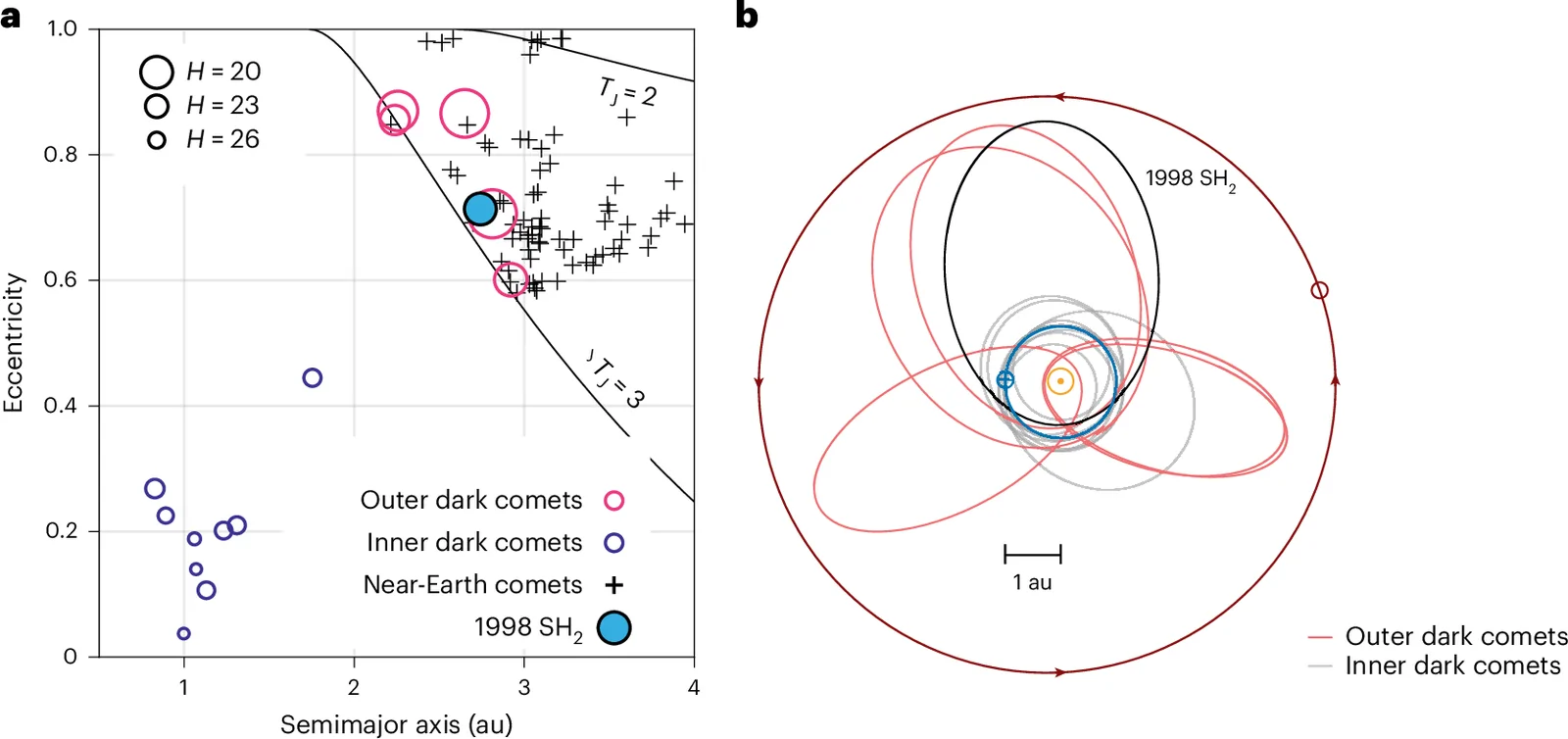
The outer dark comets, including 1998 SH_2_, are a distinct population to the inner dark comets. **a**, The distribution of eccentricity and semimajor axis for dark comets (empty circles), 1998 SH_2_ (filled circle) and Jupiter family comets (crosses). Outer and inner dark comets are clearly delineated by orbits and brightness. The size of the circles scales with the absolute magnitude *H*. The solid curves delimit the area corresponding to the definition of a Jupiter family comet where the Tisserand parameter with respect to Jupiter, *T*_j_, is between 2 and 3. **b**, Orbits of the outer dark comets (red), inner dark comets (light grey) and 1998 SH_2_ (black) compared with those of the Earth (blue) and Jupiter (cardinal). The object 1998 SH_2_ is clearly in family with the outer dark comets on a Jupiter family comet-like orbit.

Near-Earth object (875163) 1998 SH_2_ is currently classified as an asteroid as no cometary activity has been detected. However, its orbit is in line with that of other outer dark comets^18^ (Fig. 1) and its Tisserand parameter with respect to Jupiter is 2.9, which is typical of Jupiter family comets^23^. Moreover, based on infrared measurements from the Near-Earth Object Wide-field Infrared Survey Explorer (NEOWISE)^24^, 1998 SH_2_ has a diameter of 380 ± 57 m and a low albedo of 0.058 ± 0.024, which is compatible with what is observed for comets^25^ and also C-, D- and P-type asteroids^26^.

1998 SH_2_ made a close approach to Earth at a distance of 0.02 au on 30 August 2025. Monostatic radar observations^27^ were scheduled for Goldstone’s DSS-14 70-metre antenna for 26 August and 2 September 2025. The pointing for the 26 August track had a formal 3*σ* uncertainty of ±24″, based on an orbit estimated from 148 optical astrometric measurements from 17 September 1998 to 27 October 2016. 1998 SH_2_ completed two full orbits around the Sun in the timespan between the last measurement and the radar track. Unexpectedly, 1998 SH_2_ was not detected during the 26 August track within DSS-14’s 70″ primary beam width, indicating a systematic effect that had not previously been accounted for.

**Extended Data Fig. 1:**
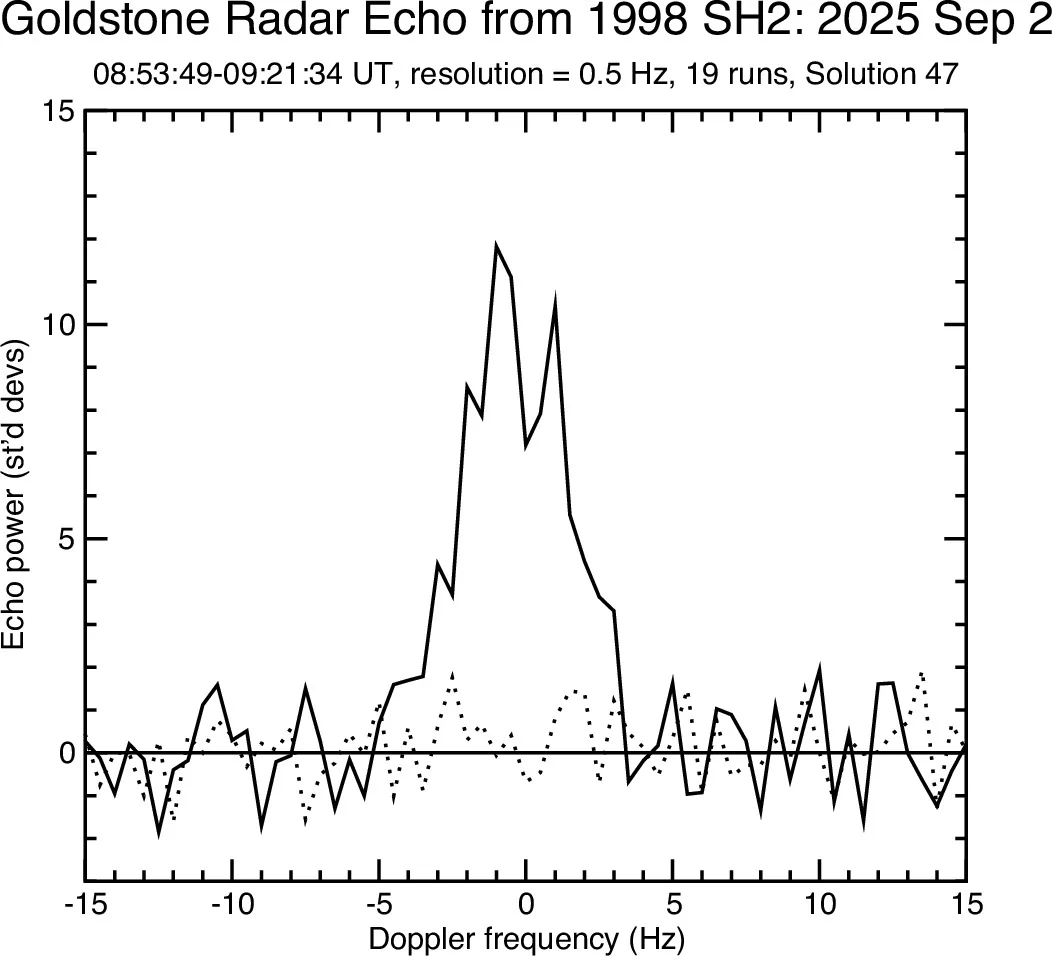
Echo power spectrum obtained on 2 September 2025, at a frequency resolution of 0.5 Hz. Echo power in standard deviations is plotted as a function of Doppler frequency. The solid line marks the opposite sense circular polarization from the transmitted signal, and the dashed line marks the same sense polarization. ‘Runs’ is the number of complete transmit and receive cycles summed in this spectrum.

On 31 August 2025, the Southern Observatory for Near Earth Asteroids Research Wykrota-Centro de Estudos Astronômicos de Minas Gerais observatory in Serra da Piedade, Brazil, recovered 1998 SH_2_, providing the first tracking data since 2016. 1998 SH_2_ was found 153″ from the gravity-only prediction, corresponding to a 19*σ* offset, which explains the failed radar detection. Adding a transverse non-gravitational acceleration *A*_2_(1 au/*r*_H_)^2^, where *r*_H_ is the heliocentric distance^28^, led to an estimate *A*_2_ = (−1.4 ± 0.1) × 10^−11^ m s^−2^, which corrected the offset and enabled a fit to the entire data arc (more details in ‘Orbital fit’ in Methods). The detected non-gravitational perturbations were then confirmed by more than 200 additional optical observations and the delay measurement from the successful radar track on 2 September 2025 (see Extended Data Table 1 and Extended Data Fig. 1 for details). The optical observations included 40 high-precision measurements with 0.1″ astrometric uncertainties that we collected with the 1.54-metre Danish Telescope (Dk154) in La Silla, Chile.

The transverse acceleration *A*_2_(1 au/*r*_H_)^2^ is generally used to model the so-called Yarkovsky effect^20^, which is due to radiative recoil of anisotropic thermal emission. As such, transverse non-gravitational acceleration via Yarkovsky measurements has been estimated on ~500 near-Earth asteroids^28^. *A*_2_ captures the dependence of the non-gravitational acceleration on the physical properties of the asteroid, for example, it is inversely proportional to the asteroid’s diameter. The largest possible *A*_2_ compatible with the Yarkovsky effect for an object of the size of 1998 SH_2_ is ~1.3 × 10^−12^ m s^−2^, which is 10 times smaller than the detected acceleration. We therefore conclude that this acceleration must be caused by another mechanism such as cometary outgassing. A nuclear radius of 170 m and this acceleration yield an estimate^16^ of the production rate (if driven by ougassing of H_2_O) of *Q*(H_2_O) ≈ 1.2 × 10^24^ molecules per second.

## Detection of 1998 SH_2_’s cometary activity

**Fig. 2:**
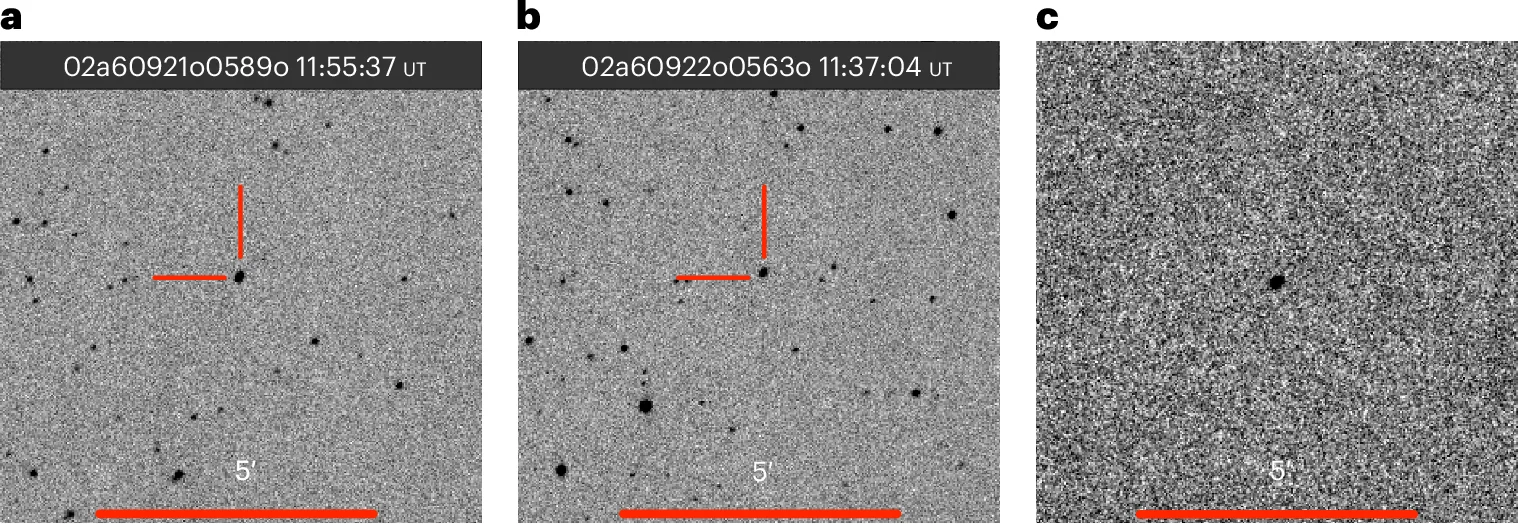
ATLAS images of 1998 SH_2_. **a**,**b**, Individual cutout o-band exposures from the ATLAS-Chile telescope taken on 3 (**a**) and 4 (**b**) September 2025. The cutouts are 9.3′ on a side (1.86*″* per pixel), with 5′ indicated. The asteroid is marked using crosshairs. The slight elongation of the asteroid is due to its motion over a 30-s ATLAS exposure. **c**, A median stack of the eight exposures from 3 and 4 September at a pixel scale of 2*″* per pixel. No coma nor tail are apparent.

To investigate the possibility that 1998 SH_2_ could be a comet, we looked for observational evidence of cometary activity. The Asteroid Terrestrial-impact Last Alert System (ATLAS)^29^ survey obtained 53 images of 1998 SH_2_ from 3 September 2025 to early October. However, no individual exposure revealed cometary activity. Figure 2 shows two individual images and a stack of 8 × 30 s observations collected by the ATLAS 0.5 m telescope on Haleakala through the ATLAS o-band filter from 3 and 4 September, with a seeing of 5″ and when the brightness of 1998 SH_2_ was *V* ≈ 15. 1998 SH_2_ looks stellar like an asteroid would.

**Fig. 3:**
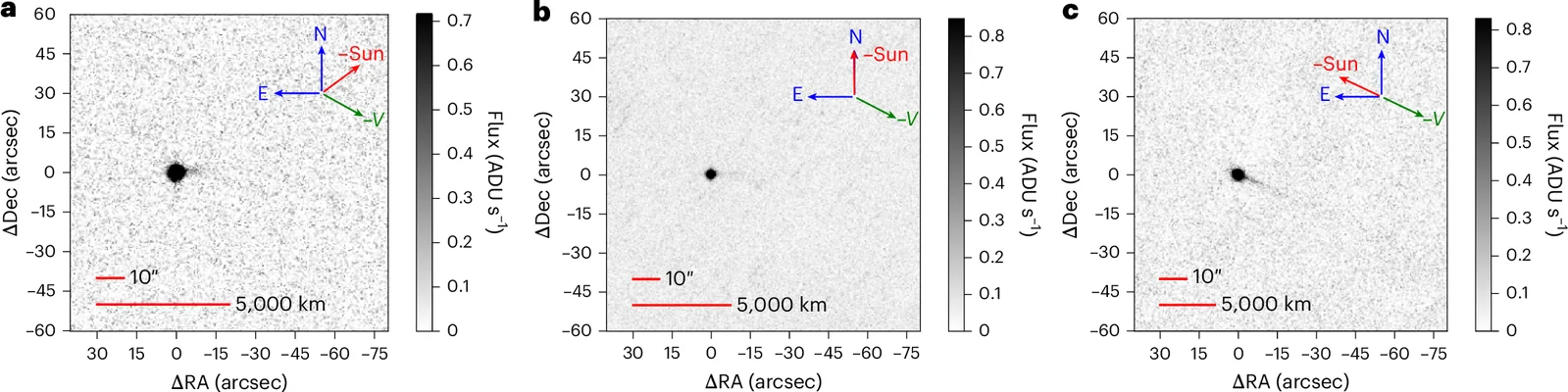
Stacked background-subtracted images of 1998 SH_2_. The linear greyscale covers the range 0 to 5*σ* of the sky noise. The arrows indicate the orientation and the projected anti-solar (−Sun) and negative heliocentric velocity (−*V*). The bars give the scale in arcseconds and kilometres. **a**, 13 September 2025, 3,600 s, R filter, Dk154. **b**, 17 September 2025, 360 s, gri filter, CFHT. **c**, 30 September 2025, 5,940 s, clear filter, VLT. Refer to Table 1 for the details. Dec, declination; RA, right ascension; ADU, analog-to-digital units.

On 17 September 2025, we obtained 3 × 120 s gri exposures of 1998 SH_2_ using the 3.6 m Canada-France-Hawai‘i Telescope (CFHT) on Maunakea, Hawai‘i, tracking non-sidereally. Although the object’s full-width at half-maximum (FWHM; 0.65″) was comparable to that of field stars (0.67″), the object displayed a low-surface-brightness tail extending west over approximately 20″. The tail was also visible in a stack of 11 × 180 s images also obtained with CFHT on 18 September (with variable seeing from 1.01″ to 1.54″), but was not detected on 23 September (6 × 180 s images, ~1.0″ seeing) or 30 September 30 (6 × 180 s images, ~0.6″ seeing), when 1998 SH_2_ was noticeably fainter as expected from the larger heliocentric and geocentric distances and illustrated by the increasing *V* magnitude derived from the ephemeris (see Table 1, which assumes a constant absolute magnitude). The 17 September 2025 images are shown in Fig. 3b.

**Table 1:** Observation circumstances of the images analysed, or otherwise used to assess whether a tail was visible

Epoch	*r*_H_ (au)	Δ (au)	*V*	Telescope	*N*_exp_	Filter	Exp (s)	Seeing (″)
3 and 4 September 2025	1.048	0.044	15.4	ATLAS	8	o	240	4.6
13 September 2025	1.144	0.139	17.3	Dk154	48	R	3,600	1.39
17 September 2025	1.189	0.184	17.9	CFHT	3	gri	360	0.66
23 September 2025	1.253	0.251	18.9	CFHT	6	gri	1,080	1.0
30 September 2025	1.325	0.332	19.8	CFHT	6	gri	1,080	0.6
30 September 2025	1.324	0.329	19.8	VLT	99	Clear	5,940	0.61

Epoch is the UT night during which the observations were obtained. *r*_H_ and Δ are the helio- and geocentric distances. *V* is the visual magnitude from the ephemerides (not a measurement). *N*_exp_ is the number of exposures. Filter is the name of the filter used. Exp is the total exposure time. Seeing is the FWHM of the background stars.

Encouraged by the CFHT detection, we obtained a deep sequence of 100 × 60 s images through a clear filter on 30 September 2025, using the 8 m European Southern Observatory (ESO) Very Large Telescope (VLT) Unit 1 (UT1) on Paranal, Chile (seeing 0.60″). The object appeared slightly broader than field stars (0.71″) and exhibited a long (>20″), narrow tail extending to the southwest (Fig. 3c). We subsequently reviewed images that we had previously taken with the Dk154 at the ESO La Silla Observatory, Chile, on 13 and 14 September 2025. A stacked series of 48 × 75 s R-filter images (1.64″ seeing) also shows the tail (Fig. 3a). The telescopes and instruments used for these observations, and the data processing steps are described in ‘Optical observations’ in Methods.

## Morphology and tail

**Extended Data Fig. 2:**
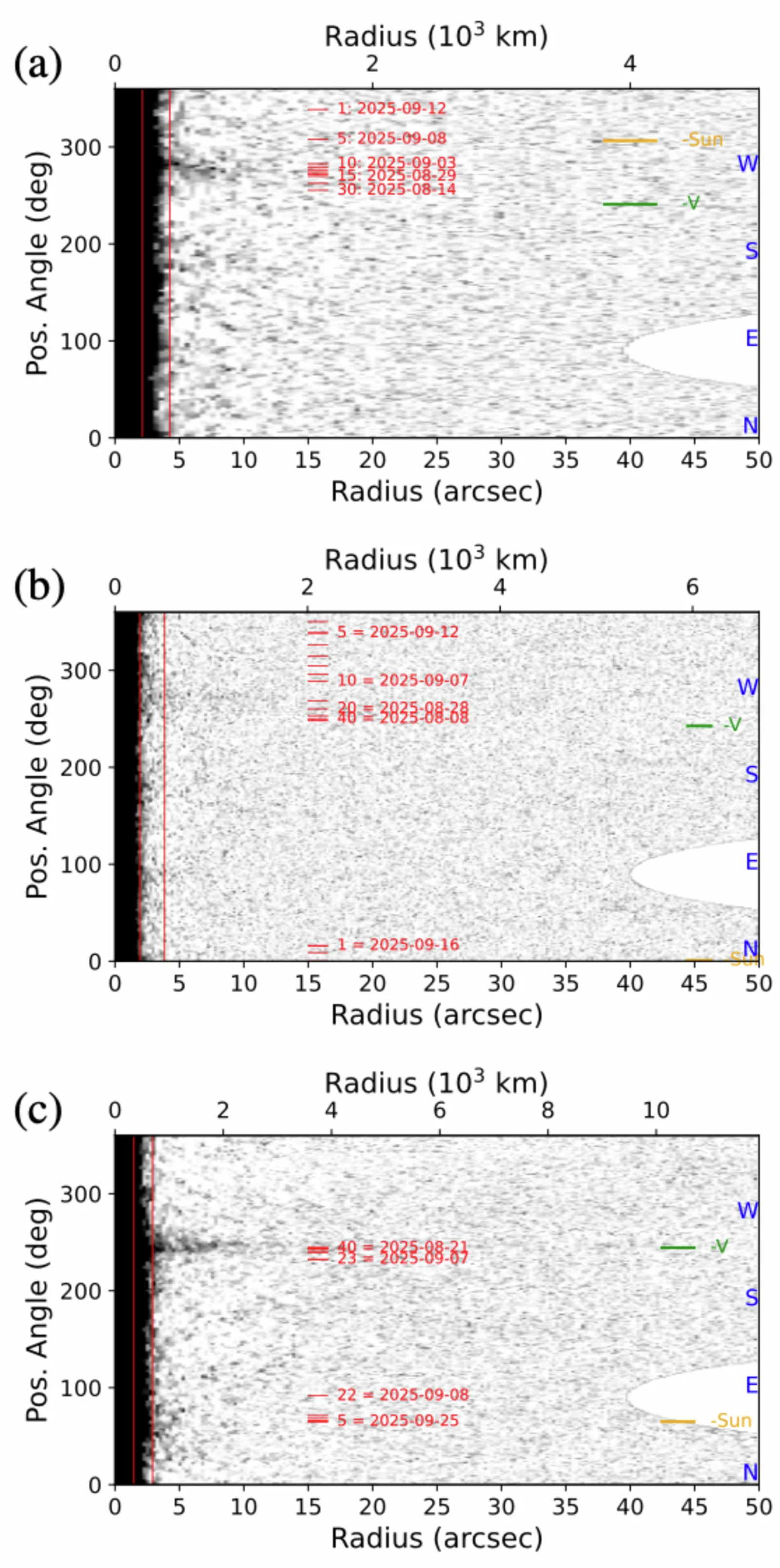
Polar projection of the images of 1998 SH_2_. The color scale is the same as Fig. 3. Position angle is measured from the North to the East. The radius is labeled in arcseconds and kilometers. The vertical red lines mark 1 and 2 HWHM seeing. The horizontal ticks indicate the orientation and the projected anti-solar and negative heliocentric velocity. The red ticks mark the position angle of some synchrones, labeled in days before the observations and corresponding UT date. **a**, 13 September 2025, 3600 s, R filter, Dk154; **b**, 17 September 2025, 360 s, gri filter, CFHT; **c**, 30 September 2025, 5940 s, clear filter, VLT. Note the narrow range of synchrones covered by the tail.

Inspection of the CFHT, VLT and Dk154 images of 1998 SH_2_ reveals a small but clearly resolved faint coma extending at least 10″ from the object. We reported the detection of cometary activity to the Minor Planet Center. As a result, 1998 SH_2_ is now a dual-status object and has received an additional comet provisional designation, P/1998 SH_2_, as well as a comet number. The coma’s contribution to the total brightness is modest, increasing from 0.06 ± 0.01 to 0.24 ± 0.01 magnitudes over the 3 epochs (13–30 September 2025; see ‘Cometary activity’ in Methods for details on these measurements). While part of this increase could be due to a rotational variability of the nucleus, this suggests a possible increase in activity during this period. Moreover, the images show a faint, narrow tail extending at least 20″ from the nucleus. Using a dust dynamics model, we converted the tail’s position angle to a dust release date (illustrated in Extended Data Fig. 2). Data from all three epochs consistently point to the tail dust being released between 1 and 7 September 2025. The images are not sensitive to dust released before or after these dates and therefore, this analysis cannot determine when the emission process started or ended.

**Extended Data Fig. 3:**
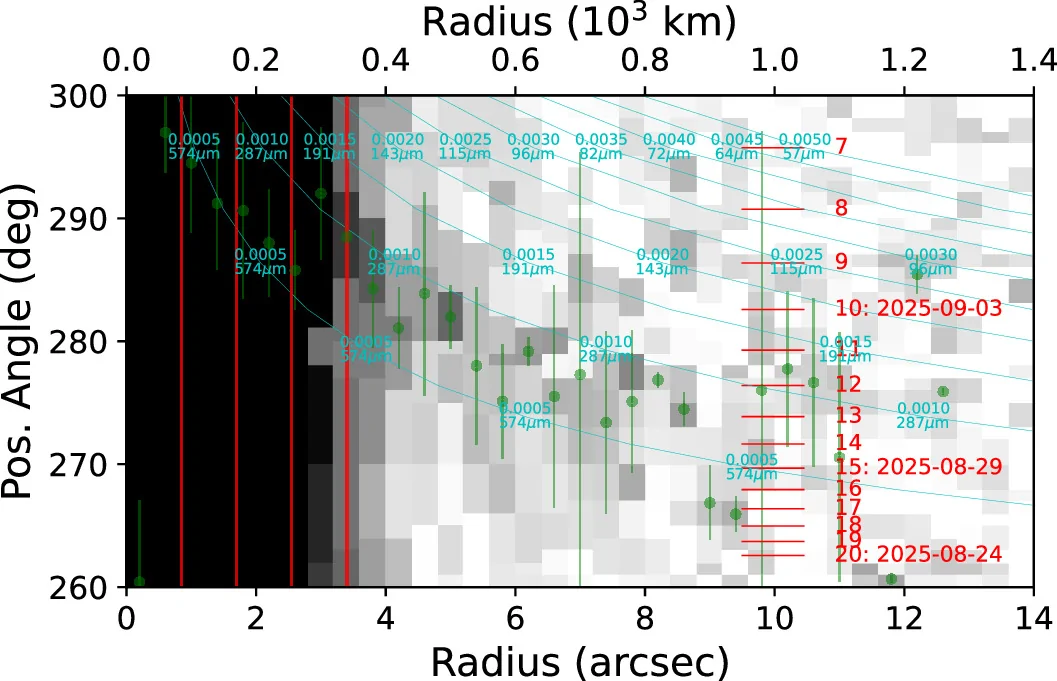
Tail area in the polar projection of the 13 September 2025, image of 1998 SH_2_. This is a close-up of Extended Data Fig. 2a.The vertical red lines mark 1 to 4 HWHM seeing. The red ticks mark the position angle of some synchrones, labeled in days before the observations. The cyan curves mark the locus of dust grains with the same size; their labels specify their values for *β* and their radii a in *μ*m. The green symbols mark the peak position angle and FWHM at that radius.

A more detailed analysis of the Dk154 image from 13 September, which offered the best spatial resolution due to the object’s closest approach to Earth, indicates that the tail is composed mostly of large grains with radius *a* ≈ 400 μm released from the nucleus between 29 August and 7 September 2025 (see ‘Analysis of the tail’ in Methods and Extended Data Fig. 3). The gas production rate we obtained is fully capable of lifting even larger grains provided that the nucleus is not considerably denser than ~1,300 kg m^−3^. Laboratory simulations suggest that large particles are ejected when ice sublimation occurs below the surface^30^.

**Extended Data Fig. 4:**
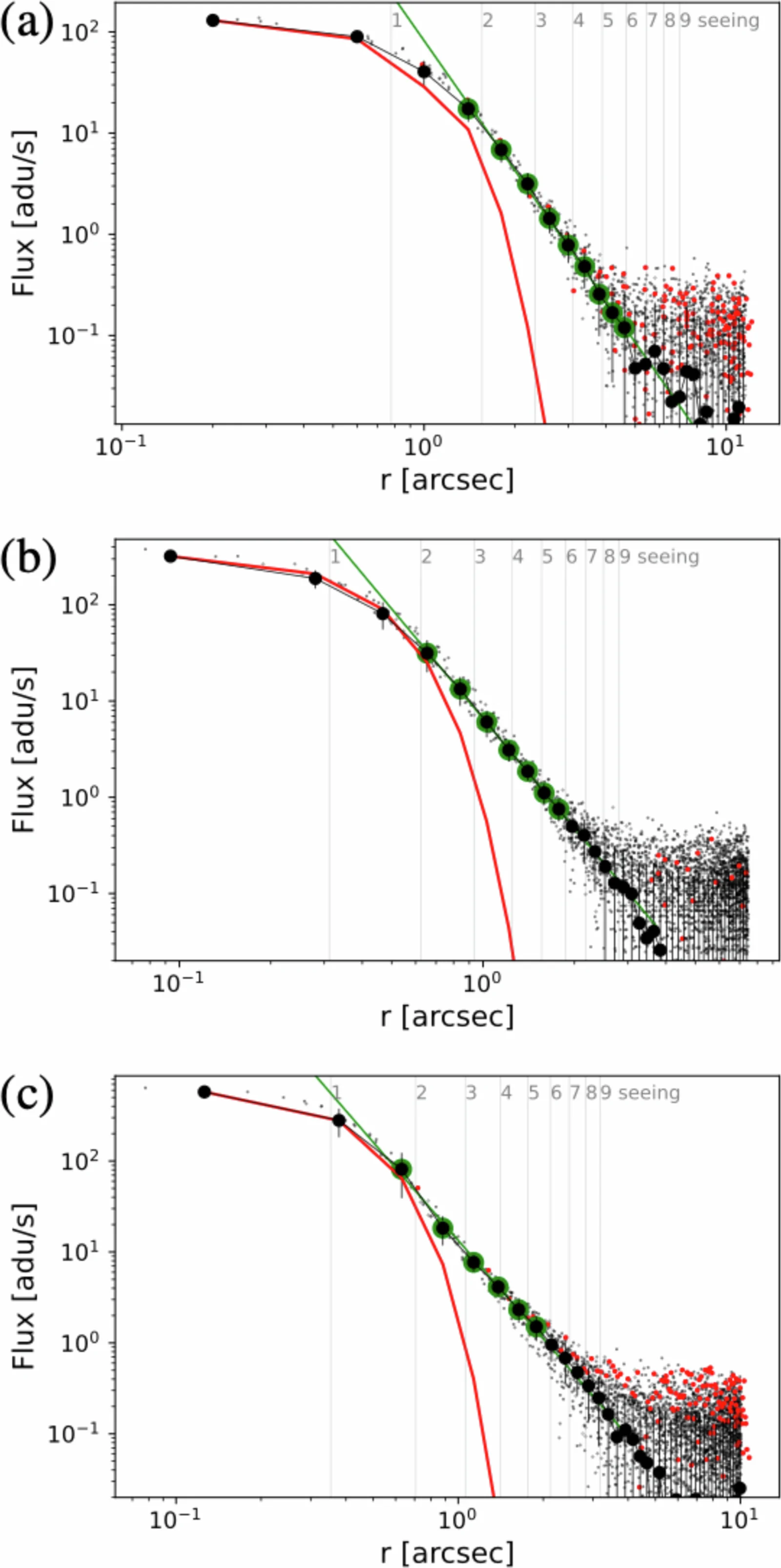
Radial profile of 1998 SH_2_, in log-log scale. The small dots correspond to individual pixels of the images from Fig. 3; those in red correspond to the position angle of the tail and were ignored in our analysis. The large, connected dots mark the median value and standard deviation in an annulus. The green line is a linear fit over the points marked in green. The red curve is a median profile of the point-spread-function measured from several field stars; the vertical grey lines mark 1–9 PSF HWHM. **a**, 13 September 2025, 3600 s, R filter, Dk154; **b**, 17 September 2025, 360 s, gri filter, CFHT; **c**, 30 September 2025, 5940 s, clear filter, VLT.

Collectively, the images demonstrate that dust release took place continuously over the late August to late September 2025 period, and do not imply that the activity was confined to that range. Rather, this suggests a sublimation-caused cometary activity process and firmly precludes instantaneous processes (such as a small impact) or repetitive processes (such as rotation-induced shedding), which would produce distinct morphological signatures. Furthermore, the object’s surface brightness, shown in Extended Data Fig. 4, decreases with distance *r* from the nucleus as *r*^−*n*^, with a steep exponent *n* decreasing from 4.3 to 3.6 over the course of the observations. For an object releasing dust at a constant rate, the expected value is *n* = 1, increasing to *n* = 1.5 if solar radiation pressure materially affects dust evolution^31,32^. A steeper slope (*n* > 1.5) can be caused by the destruction of grains over time or by an increase in the activity level.

The object passed perihelion on 21 July 2025, weeks before the observations. Therefore, the increase of activity between the beginning and the end of September took place several weeks after perihelion. Comet outgassing is often asymmetric around perihelion. A complex interplay between the nucleus shape, rotation state and thermal processing governs the onset and evolution of cometary activity. In general, outgassing is highly variable and does not follow a universal pattern ^33^. This asymmetry can also be driven by low thermal conductivity of the body, causing a lag between the incoming radiation and the heat reaching subsurface volatile ices. For instance, in the case of comet 67P/Churyumov–Gerasimenko, a lag of several months has been estimated^34^ at a depth of 0.5 m. The post-perihelion delay of 1998 SH_2_’s activity therefore support the hypothesis that the sublimation took place below the surface.

## Implications for the small-body population

1998 SH_2_ is the first object for which cometary activity was initially predicted based on non-gravitational perturbations to its motion and then confirmed by targeted observational data. The astrometric observation arc from 1998 to 2025 enables the detection of non-gravitational perturbations to the object’s motion, and favourable observing conditions following the 30 August 2025 close approach and access to large-aperture telescopes enabled the detection of cometary activity. This result suggests that also the other outer dark comets, which have similar size and orbital properties to that of 1998 SH_2_, could be confirmed as active. While these objects have sufficient arc lengths to detect non-gravitational perturbations, the lack of sufficiently sensitive observations during favourable observing opportunities has prevented the observational confirmation of their cometary activity.

More broadly, as of late 2025 there are 2009 known near-Earth asteroids with a Jupiter Tisserand parameter between 2 and 3, the range corresponding to Jupiter family comets. Theoretical predictions suggest that a fraction of near-Earth objects with a Tisserand parameter in that range may be nearly extinct comets^35^. If a substantial fraction of these are truly comets and contain volatiles, there could be important ramifications for the delivery of water to the Earth by an analagous population of volatile-bearing near-Earth objects present when the Earth was forming^36–39^. Targeted searches for activity as in this paper or as done for (3552) Don Quixote^40^ or main-belt comets^41^, and the advent of Vera Rubin Observatory’s operations^42^ may reveal that more objects with these orbital properties are outgassing. In particular, 285 of these objects, including 1998 SH_2_, are classified as potentially hazardous asteroids, meaning that their orbits come within 0.05 au of the Earth’s orbit, and their sizes are estimated to be at least 140 m. These are the most promising candidates for future detections of activity because their proximity to Earth allows for higher-resolution imaging and better constraints on non-gravitational perturbations through orbit fitting.

Small-body surveys observe the sky to find near-Earth objects and enable mitigation efforts in case an object were found to be on a collision course. While the probability of an Earth impact for 1998 SH_2_ over the foreseeable future remains zero even after the detection of non-gravitational perturbations, this may not be the case for other potentially hazardous asteroids that turn out to be comets. Because non-gravitational perturbations due to outgassing are larger than radiation-based ones, the future trajectory of these objects may be more uncertain than currently assumed. Fully capturing the resulting uncertainties may lead to a different assessment of any possibility of future impact.

Our discovery of 1998 SH_2_ displaying cometary activity has broader implications for planetary defence. The possibility that a number of potentially hazardous objects currently classified as asteroids could turn out to be comets could increase the relative Earth impact risk from comets with respect to asteroids. The different physical properties of comets, for example, composition, directly affect the design of any deflection mission, such as the recent Double Asteroid Redirection Test^43^. Moreover, models of the actual impact effects^44^ if an object were to hit Earth can lead to different assessments based on the nature of the object. Therefore, planetary defence researchers should consider comet properties more consistently when evaluating the risk of and planning for impact threats.

Finally, our findings are relevant to the theoretical interpretations for the presence of non-gravitational acceleration affecting the motion of interstellar object 1I/‘Oumuamua despite the lack of detected cometary activity^11–13^. This puzzle led to a variety of hypotheses, including theories that invoke (1) radiation pressure^45–47^ or (2) comet-like outgassing^48–51^ for the driver of the non-gravitational acceleration.

## Methods

### Orbital fit

The orbital solution was estimated through a least-squares fit^52^ to the available optical and radar astrometry. We corrected optical astrometry to remove star catalogue biases^53^ and weighted data according to a statistical analysis of past performance for the different observatories^54^. Our own Dk154 and radar observations were weighted based on the individual measurement uncertainty. The parameters estimated from the fit are the cometary orbital elements and the *A*_2_ parameter used to model non-gravitational perturbations as a transverse acceleration *A*_2_*g*(*r*_H_) where *g* provides the functional dependence on the heliocentric distance *r*_H_ (ref. ^55^). In this paper, we set *g*(*r*_H_) = (1 au/*r*_H_)^2^, which is the typical choice to model the Yarkovsky effect^28^.

### Optical observations

In early September 2025, 1998 SH_2_ was imaged during routine observations by the ATLAS survey^29^. Typical ATLAS survey exposures are sidereally tracked and 30-s long, using either the ATLAS o (~*r* + *i*) or c (~ *g* + *r*) filter, with 4 exposures taken over a 30-min interval at the same footprint on the sky. The ATLAS projected pixel scale is 1.86″ on sky.

The Dk154 observations were obtained with the Danish Faint Object Spectrograph and Camera (DFOSC)^56^, using either the Cousins (Bessell) R filter or no filter. The detector is an E2V231-42, with a pixel scale of 0.396″ on sky. The telescope was tracked at a half of the apparent rate of the observed target, resulting in same trailing of field stars and the target.

The CFHT observations were obtained with the MegaPrime instrument^57^, using the ‘gri.MP9605’ filter. While MegaPrime is a mosaic of 40 charge-coupled devices (CCDs), the detector used was ‘ccd23’, a Marconi/EEV CCD with a 0.187″ pixel scale.

The VLT observations were obtained with the Focal Reducer/low dispersion Spectrograph 2 (FORS2)^58^, using the clear filter, optimized for throughput. FORS2 is equipped with a 1 × 2 mosaic of CCDs. We used ‘chip 1’, an MIT/LL detector, with a pixel 0.126″ pixel read binned 2 × 2 resulting in a 0.252″ on sky. The observation circumstances are listed in Table 1.

### Image processing

The data were processed using standard electronic bias subtraction and flat-fielding with averaged twilight or dome exposures. Images from the Dk154 and VLT were aligned with subpixel accuracy using the centroids of tens of background stars. Stars were identified using Source Extractor^59^ in the SEP implementation^60^ and cross-matched with Astroalign^61^, resulting in the accurate offset between frames. The star-aligned frames were then stacked using a sigma-clipping median rejection, creating a deep star background reference image. The object was rejected by the median due to its motion and is thus absent from this reference. In the case of CFHT, as only three frames were available, a master background stack could not be produced. The stars and background objects were therefore manually masked in the three frames.

The ATLAS exposures were reduced using its standard image reduction pipeline^29^. Transient sources were detected by subtracting the ATLAS o-band template image, and the asteroid was detected by linking catalogue detections using the ATLAS Moving Object Processing System. Eight of the subtracted images from 3 and 4 September 2025 were registered to a pixel scale of 2″ per pixel and further stacked using a ‘weighted median’ (50% quantile of cumulative weights) to search for a signal of cometary activity.

The ephemeris of 1998 SH_2_ was retrieved from Jet Propulsion Laboratory (JPL)’s Horizons^62^ for each frame’s epoch. Using the previous inter-frame offsets and the reference image’s astrometric calibration, the necessary offsets to align the images on 1998 SH_2_ were computed. The star-aligned reference image was also subtracted from the individual frames. This removed the extended signal (for example, stellar point spread function (PSF) wings and galaxies), leaving only small residuals near the star cores due to minor seeing variations, subpixel misalignment and VLT diffraction pattern rotation. These residual frames were then shifted and stacked. The sigma-clipping median rejected background star residuals, cosmic rays and blemishes, resulting in deep stacks showing the object on a clean, empty background (Fig. 3).

### Cometary activity

The surface-brightness profile of the object was computed using the final stacks from Fig. 3, and compared with the profile of the PSF, evaluated by averaging several well-exposed field stars in the corresponding master background stack.

The flux was integrated in a series of circular annuli centred on either the object or the star template. The error was taken as the standard deviation of the individual pixels within each annulus. For the PSF profile of trailed images, we only used pixels in angular regions perpendicular to the star’s trailing direction. For the object’s profile, we rejected pixels within 5° of the tail’s position angle to avoid contamination. The PSF profile was normalized so that its peak flux matched that of the object. The resulting profiles are shown in Extended Data Fig. 4.

1998 SH_2_ shows a clear flux excess over the PSF profile, extending beyond 10″. Quantitatively, this excess represented 0.06 ± 0.01, 0.210 ± 0.008 and 0.241 ± 0.008 mag for the Dk154, CFHT and VLT observations, respectively. The error bars seem small in absolute terms, but one must keep in mind that this is a relative measurement to the stellar profile. The total duration of each observation is unlikely to average out the possible rotational variability of the object, which could be in the 0.2–0.4 range^63^ for such a small object. An intrinsic variation of the nucleus of 0.2 mag would cause a change of the (unchanged) coma contribution of ±0.04 mag. The overall increase from 0.06 to 0.2 is therefore likely significant, but the variation from 0.21 to 0.24 could be caused by the nucleus rotation.

At radii *r* beyond the seeing disk, the excess flux follows a linear trend in the log–log plot, corresponding to a surface-brightness profile that evolves as *r*^−*n*^. A linear regression performed over 2 ≤ *r* ≤ 4 half-width at half-maximum yielded exponents *n* = 4.3, 3.8 and 3.6 for Dk154, CFHT and VLT, respectively. These values are considerably steeper than the *n* = 1 expected for a steady-state, isotropic coma^31^ or the *n* = 1.5 maximum expected when including the effect of solar radiation pressure^32^. Such steep slopes can be caused by the sublimation of icy grains over time, or by an increase in activity. Given that the fraction of extended flux relative to the total flux was possibly increasing with time, we favour the interpretation of increasing activity, with the rate of increase becoming slower with time.

In summary, the object was active on 13, 17 and 30 September 2025, and its activity level was possibly increasing throughout this period.

### Analysis of the tail

The 1998 SH_2_ tail morphology was analysed using the Finson–Probstein method^64^, which models the motion of dust grains under the effects of solar gravity and solar radiation pressure. This analysis generates families of synchrones, connecting particles emitted at the same time, and syndynes, connecting particles with the same *β*, the ratio of the radiation pressure to the solar gravity. On the images, the synchrones appear as radial lines whose position angle (PA) is related to time of dust emission. To visualize and analyse these, the images were transformed to polar coordinates, shown in Extended Data Fig. 2, in which the nucleus appears as the broad bright band at low radii, and the tail as an horizontal feature.

The Dk154 image indicates that the dust in the tail was released between 30 August and 7 September 2025. The shallower CFHT data suggest a release window between 28 August and 7 September. Owing to Earth’s position nearly in 1998 SH_2_’s orbital plane during the VLT observations, the synchrones nearly collapse into a single line: at PA ≈ 65° for dust emitted after 9 September, and at PA ≈ 244° for pre-7 September emissions (matching the PA of the projected negative velocity vector). The entire observed tail lies along this position angle. In summary, the tail in each image corresponds to the same continuous activity event that took place between 30 August and 7 September 2025.

Although the Dk154 image had worse seeing, the closer proximity of 1998 SH_2_ to Earth provided the highest-spatial-resolution look at the object. Critically, the tail reveals additional information via the Finson–Probstein syndynes. The *β* value is related to the grain radius *a* (m) and density *ρ* (kg m^−3^) via:

1 $$\beta =5.74\times 1{0}^{-4}\frac{Q}{\rho a},$$

where *Q* ≈ 1 is the radiation pressure efficiency, which depends on the grain material^64^. While *ρ* can vary widely—from 1,000 kg m^−3^ (a traditional cometary value) to 1,900 ± 1,100 kg m^−3^ from in situ measurements^65^ on comet 67P, and up to 3,000 kg m^−3^ for S-type asteroids^66^—we adopt *ρ* = 2,000 kg m^−3^ for this analysis. Extended Data Fig. 3 shows a subset of the Dk154 image from Extended Data Fig. 2, with *β* and the corresponding radius *a* labelling the plotted syndynes. The PAs of the synchrones are also marked. To characterize the tail’s shape, a Gaussian profile was fitted to the tail at various distances from the nucleus, and its central PA and FWHM are marked as green symbols.

The peak of the tail is confined within the 0.0005 ≤ *β* ≤ 0.0010 range, corresponding to emission times from 29 August until 7 September 2025. The tail is too faint and diffuse to measure its position before 29 August with this method, and it is too close to the nucleus and lost in its glare after 7 September, but there is no indication of an abrupt change. The measured range of *β* corresponds to very large grains, on the order of 400 μm. Cometary dust grains typically follow a power-law size distribution^67^ with an index of about −4, and up to an upper limit *a*_max_. The fact that no grains appear below the *β* = 0.0005 syndyne suggests an upper size limit of *a*_max_ ≈600 μm. As grain brightness scales as *a*^2^, the observed grains follow a brightness power-law distribution with an index of about −2. This implies that grains smaller than ~400 μm should be more numerous and brighter, which is not the case. This discrepancy strongly suggests that the actual grain size distribution is narrowly limited to the 300–600 μm range.

From the gas production rate computed above, ${Q}_{{{\rm{H}}}_{2}{\rm{O}}}\approx 1.2\times 1{0}^{24}$ molecules per second, we estimate the largest dust grain that can be lifted from the nucleus. For that critical radius, the gas drag equals the weight of the grain. Assuming a grain density of 1,000 kg m^−3^ and a nucleus density of 500–1,000 kg m^−3^, the critical radius can be estimated^68^ as *a* ≈ 1.6 mm, confirming that the large grains observed can easily be lifted by the gas. Alternatively, assuming that the largest grains observed, with *a* ≈ 600 μm, correspond to the critical radius, the density of the nucleus would be ~1,300 kg m^−3^. We can therefore use this value as an upper limit to the nucleus density.

While typical cometary dust is in the micrometre range, very large grains (up to centimetere scale) have been detected. Such large grains were observed remotely, for instance^69,70^, near comet C/2001 A_2_ or in situ near comet 67P. Laboratory simulations suggest that these large particles are ejected when ice sublimation occurs below the surface, leading to a buildup of pressure that explosively expels the material^30^. A fast rotation could also contribute to the ejection of large particles, for example, similar to one of the hypotheses formulated for 133P/Elst–Pizarro^71^. Notwithstanding the origin of the grains, they were continuously released from 29 August until 7 September 2025, with no indication of an abrupt start or stop at either these dates.

### Radar

Radar observations of 1998 SH_2_ occurred at Goldstone (8,560 MHz, 3.5 cm) on 26 August and 2 September 2025, dates that straddled the closest approach within 0.02 au on 31 August, when the asteroid was too far south for Goldstone to track. The 26 August observations did not produce a detection but observations on 2 September were successful. The radar observations used standard data acquisition and reduction techniques^72,73^. At the time of the observations, problems with one of the klystron amplifiers limited the transmitter power to 240 kW, or slightly more than one-half of the nominal value.

On 26 August, we estimated that signal-to-noise ratios (SNRs) would be strong enough to obtain an echo within a few minutes. Given the diameter, and the fact that nearly all near-Earth asteroids (NEAs) >0.15 km in diameter have rotation periods slower than 2.1 hours, we expected an echo bandwidth of less than about 20 Hz. After observing for about 20 minutes without detecting an echo, we checked different frequency resolutions in case the echo was much narrower or wider than expected. After 40 minutes, there was still no echo, so we abandoned 1998 SH_2_ and observed a different asteroid. Earlier during the observing session on 26 August, we detected radar echoes from 1997 QK_1_, and after we stopped the 1998 SH_2_ observations, we also detected echoes from 2025 QX_4_, so we knew that the radar system was functioning well and suspected that the pointing was off for 1998 SH_2_, which was later confirmed after the observations on 26 August concluded.

We began on 2 September with continuous-wave observations and saw an echo within 2 minutes. The echo has a bandwidth of 7 Hz and is centred on the Doppler frequency predicted by the ephemeris (Extended Data Fig. 1). We then transmitted coded waveforms with time delay resolutions of 10 μs, 11 μs and 1 μs (distance resolutions of 1,500 m, 1,650 m and 150 m) to estimate the range. The entire sequence of Goldstone radar observations spanned about 67 min and is summarized in Extended Data Table 1.

On the basis of infrared data obtained by the NEOWISE mission, the diameter and albedo of 1998 SH_2_ are estimated^24^ as of 380 ± 60 m and 0.058 ± 0.024. The width of a radar echo is given by:

2 $$B=\frac{4{\rm{\pi }}D\cos (\delta )}{\lambda P},$$

where *B* is the bandwidth or Doppler broadening of the echo, *D* is the diameter, *δ* is the subradar latitude, *λ* is the wavelength and *P* is the rotation period. If the rotation period is known, then equation (2) constrains the pole-on extent of the asteroid. For 1998 SH_2_, a rotation period has not been reported but the bandwidth, diameter and equation (2) allow us to estimate the period. Given the bandwidth of 7 Hz and a diameter of 380 m, equation (2) places an upper bound on the rotation period of 5.4 h under the assumption that the diameter is correct and that 1998 SH_2_ is not considerably elongated.

The echo in Extended Data Fig. 1 shows a dip at frequencies near the middle of the echo that is consistent with a concavity, but due to the relatively weak SNRs, the dip is also consistent with receiver noise. The narrow spikes also resemble echoes seen from satellites of binary systems, where the broad echo is from the primary and the narrow echo is from the secondary. To check, we processed the continuous-wave data at different frequency resolutions but did not find convincing evidence for a companion. We also checked the 1 μs ranging data by processing it at four different frequency resolutions and summing all the runs. Radar observations of binary NEAs observed previously at this delay resolution often show echoes from two separate objects. The summed images at 1.0 μs × 0.5 Hz resolution show a small number of pixels with SNRs ~3.5 in delay-Doppler locations expected for an object in orbit relative to the main echo, but the pixels are also consistent with noise (we expect ~30 noise pixels this strong), so the evidence for a satellite, although intriguing, is not convincing.

We searched for rotational variations in the bandwidths and spectral shapes by summing groups of 5 runs (spanning about 5 min each) processed at 0.5 Hz resolution. We did not see any variations that are statistically significant, so evidently the bandwidth did not change significantly over an interval of 1.06 h.

For 1998 SH_2_, we estimate a circular polarization ratio, that is, the ratio of the echo power in the same circular (SC) polarization state to that in the opposite circular (OC) polarization state, of SC/OC = 0.09 ± 0.03, which is lower than the average of ~0.3 seen for hundreds of other NEAs observed with radar^74,75^. This ratio is also lower than those observed for (433) Eros (0.28 ± 0.06), (25143) Itokawa (0.27 ± 0.04), (4179) Toutatis (0.29 ± 0.01), (101955) Bennu (0.18 ± 0.01) and (65803) Didymos (0.20 ± 0.02), which have been visited by spacecraft. The circular polarization ratio of 1998 SH_2_ could indicate that the near-surface is less rugged at decimetre spatial scales than the surfaces of the asteroids imaged by missions. However, modelling results^76,77^ indicate that surface texture and composition also strongly influence circular polarization ratios so roughness is not the only possibility. The low ratio of 1998 SH_2_ is inconsistent with those seen for V-, E- and some X-class NEAs (SC/OC > 0.6). The ratio is consistent with values estimated for the other spectral types, particularly a small sample of M types, and also with the lower end of the distribution for optically dark BC types and bright SQ types. The circular polarization ratio has been measured for 8 comets^78–82^ and ranges between 0.105 and 0.59; 1998 SH_2_’s value is lower than this range.

Radar echoes from some comets show a wide ‘skirt’ caused by centimetre- to decimetre-sized coma particles surrounding the nucleus. Coma echoes have been seen in radar echoes of numerous comets that were very active (for example, C/1996 B_2_ (Hyakutake)), but are not always detected from some comets that show a coma at optical wavelengths. We searched but do not see a wide coma echo for 1998 SH_2_, which is consistent with the low level of activity observed in the optical images. Thus, we conclude that cometary activity by 1998 SH_2_ on 2 September was too low to detect with radar observations at Goldstone.

The 1-μs echo occupies 2 rows, and given that the radar could illuminate only ~1/2 of the surface if the object were a sphere, this establishes that the diameter of 1998 SH_2_ is <600 m, a result that is consistent with the value of 380 m from NEOWISE^24^. The diameter of 380 m also indicates that 1998 SH_2_ is the smallest comet ever observed by radar. We used echo power spectra processed at 0.5-Hz resolution (Extended Data Fig. 1) to estimate a radar cross-section of 0.0048 km^2^ ± 35%, where the uncertainty accounts for systematic pointing and calibration errors. If we adopt the diameter of 380 m, then we obtain a radar albedo of ~0.04, which is lower than most observed among NEAs but overlaps many estimated for comet nuclei^80^. The radar albedo is a function of the near-surface bulk density^80,83,84^ and a value of 0.04 suggests a surface with porosity that is not highly compacted. The implication is that the radar albedo more closely resembles those seen from comet nuclei than from NEAs.

### Meteoroid stream

Given that 1998 SH_2_ closely approaches Earth, the possibility of a meteoroid stream giving rise to a meteor shower does exist. Assuming any meteoroid ejection occurs at relatively low velocity, a meteoroid stream would be expected to move in parallel to 1998 SH_2_. On 30 August 2025, when the orbit of 1998 SH_2_ is 0.02 au from Earth, any potential shower would appear to originate from the geocentric radiant *α*_g_ ≈ 172.3°, *δ*_g_ ≈ −0.1°, with a geocentric speed *v*_g_ ≈ 17.2 km s^−1^. However, as this date corresponds to the start of the current activity, any meteoroids that may have been observed must have been released during previous activity. Also, while ~400 μm meteoroids would produce optical meteors, this radiant is close to the helion direction, and thus, probably would be only visible by meteor radars. An in-depth simulation to better model any potential meteoroid stream is beyond the scope of our work here.

## Figures and Tables

**Extended Data Table 1:**
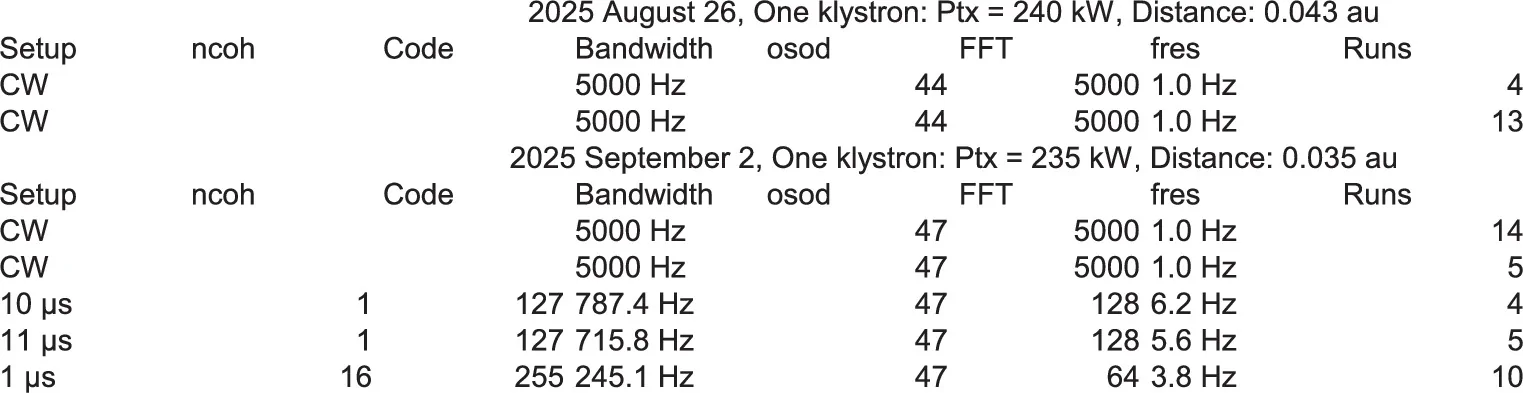
Technical details of the Radar Observations of 1998 SH_2_

The first column indicates the observing configuration, ncoh gives the number of coherent sums used to process the data, Code gives the length of the binary phase code for delay-Doppler observations used to estimate the range, OSOD refers to the orbital solution number used with our On-Site Orbital Determination software, FFT gives the fast Fourier Transform length, fres is the frequency resolution, and Runs indicates the number of transmit-receive cycles. Start UTC and Stop UTC refer to the beginning and end of data reception.

## Data Availability

The optical astrometry is available from the Minor Planet Center (https://www.minorplanetcenter.net/) and the radar astrometry is available from the Jet Propulsion Laboratory (https://ssd.jpl.nasa.gov/sb/radar.html). The ATLAS images are available on the Small Bodies Node of the Planetary Data System (https://pds-smallbodies.astro.umd.edu/data_sb/missions/atlas/index.shtml). The VLT data are available from the ESO Science Archive Facility (http://archive.eso.org/wdb/wdb/eso/eso_archive_main/query?max_rows_returned=300&ob_id=4582577%20∣%204582574). The CFHT images will become available from the Canadian Astronomy Data Centre (https://www.cadc-ccda.hia-iha.nrc-cnrc.gc.ca/en/cfht/) as soon as the 2025B semester becomes public.

## Code availability

The Jet Propulsion Laboratory orbit fitting code is proprietary but the results can be reproduced using publicly available software packages such as Monte (https://montepy.jpl.nasa.gov/), OrbFit (https://adams.dm.unipi.it/orbfit/), Find_Orb (https://www.projectpluto.com/find_orb.htm) and GRSS (https://github.com/rahil-makadia/grss).

## References

[1] Lissauer, J. J. & De Pater, I. *Fundamental Planetary Science: Physics, Chemistry and Habitability* (Cambridge Univ. Press, 2019).

[2] Jewitt, D. & Hsieh, H. H. in *Comets III* (eds Meech, K. J. et al.) 767–798 (Univ. Arizona Press, 2024).

[3] Jewitt, D. The active asteroids. *Astron. J.* **143**, 66 (2012).

[4] Hsieh, H. H. Asteroid–comet continuum objects in the Solar System. *Philos. Trans. R. Soc. A* **375**, 20160259 (2017). 10.1098/rsta.2016.0259 PMC5454227 28554978

[5] Meech, K. J. et al. Inner Solar System material discovered in the Oort cloud. *Sci. Adv.* **2**, e1600038 (2016). 27386512 10.1126/sciadv.1600038 PMC4928888

[6] Asher, D. J., Bailey, M. E., Hahn, G. & Steel, D. I. Asteroid 5335 Damocles and its implications for cometary dynamics. *Mon. Not. R. Astron. Soc.* **267**, 26 (1994).

[7] Jewitt, D. A first look at the Damocloids. *Astron. J.* **129**, 530–538 (2005).

[8] Licandro, J. et al. The visible and near-infrared spectra of asteroids in cometary orbits. *Astron. Astrophys.* **618**, A170 (2018).

[9] Borisov, G. et al. Comet C/2019 Q4 (Borisov). *Cent. Bur. Electron. Telegr.* **4666**, 1 (2019).

[10] Seligman, D. Z. et al. Discovery and preliminary characterization of a third interstellar object: 3I/ATLAS. *Astrophys. J. Lett.* **989**, L36 (2025).

[11] Meech, K. J. et al. A brief visit from a red and extremely elongated interstellar asteroid. *Nature* **552**, 378–381 (2017). 29160305 10.1038/nature25020 PMC8979573

[12] Jewitt, D. et al. Interstellar interloper 1I/2017 U1: observations from the NOT and WIYN telescopes. *Astrophys. J. Lett.* **850**, L36 (2017).

[13] Ye, Q.-Z., Zhang, Q., Kelley, M. S. P. & Brown, P. G. 1I/2017 U1 (‘Oumuamua) is hot: imaging, spectroscopy, and search of meteor activity. *Astrophys. J. Lett.* **851**, L5 (2017).

[14] Micheli, M. et al. Non-gravitational acceleration in the trajectory of 1I/2017 U1 (’Oumuamua). *Nature* **559**, 223–226 (2018). 29950718 10.1038/s41586-018-0254-4

[15] Yeomans, D. K., Chodas, P. W., Sitarski, G., Szutowicz, S. & Królikowska, M. in *Comets II* (eds Festou, M. C. et al.) 137–151 (Univ. Arizona Press, 2004).

[16] Farnocchia, D. et al. (523599) 2003 RM: the asteroid that wanted to be a comet. *Planet. Sci. J.* **4**, 29 (2023).

[17] Seligman, D. Z. et al. Dark comets? Unexpectedly large nongravitational accelerations on a sample of small asteroids. *Planet. Sci. J.* **4**, 35 (2023).

[18] Seligman, D. Z. et al. Two distinct populations of dark comets delineated by orbits and sizes. *Proc. Natl Acad. Sci. USA* **121**, e2406424121 (2024). 39652762 10.1073/pnas.2406424121 PMC11665882

[19] Vokrouhlický, D. & Milani, A. Direct solar radiation pressure on the orbits of small near-Earth asteroids: observable effects? *Astron. Astrophys.* **362**, 746–755 (2000).

[20] Vokrouhlický, D., Bottke, W. F., Chesley, S. R., Scheeres, D. J. & Statler, T. S. in *Asteroids IV* (eds Michel, P. et al.) 509–531 (Univ. Arizona Press, 2015).

[21] Farnocchia, D. et al. Radiation forces and trajectory of Hayabusa2*♯* target 1998 KY_26_. *Astrophys. J. Lett.* **993**, L9 (2025).

[22] Zhang, Q. et al. Covertly active comet (139359) 2001 ME1. *Planet. Sci. J.* **7**, 31 (2026).

[23] Duncan, M., Levison, H. & Dones, L. in *Comets II* (eds Festou, M. C. et al.) 193–204 (Univ. Arizona Press, 2004).

[24] Masiero, J. R. et al. NEOWISE reactivation mission year three: asteroid diameters and albedos. *Astron. J.* **154**, 168 (2017).

[25] Lamy, P. L., Toth, I., Fernandez, Y. R. & Weaver, H. A. in *Comets II* (eds Festou, M. C. et al.) 223–264 (Univ. Arizona Press, 2004).

[26] Mahlke, M., Carry, B. & Mattei, P.-A. Asteroid taxonomy from cluster analysis of spectrometry and albedo. *Astron. Astrophys.* **665**, A26 (2022).

[27] Benner, L. A. M., Busch, M. W., Giorgini, J. D., Taylor, P. A. & Margot, J. L. in *Asteroids IV* (eds Michel, P. et al.) 165–182 (Univ. Arizona Press, 2015).

[28] Farnocchia, D. et al. Near Earth asteroids with measurable Yarkovsky effect. *Icarus* **224**, 1–13 (2013).

[29] Tonry, J. L. et al. ATLAS: a high-cadence all-sky survey system. *Publ. Astron. Soc. Pac.* **130**, 064505 (2018).

[30] Laufer, D., Pat-El, I. & Bar-Nun, A. Experimental simulation of the formation of non-circular active depressions on Comet Wild-2 and of ice grain ejection from cometary surfaces. *Icarus* **178**, 248–252 (2005).

[31] Gehrz, R. D. & Ney, E. P. 0.7- to 23-μm photometric observations of P/Halley 1986 III and six recent bright comets. *Icarus* **100**, 162–186 (1992).

[32] Jewitt, D. C. & Meech, K. J. Surface brightness profiles of 10 comets. *Astrophys. J.* **317**, 992 (1987).

[33] Combi, M. R., Mäkinen, T. T., Bertaux, J.-L., Quémerais, E. & Ferron, S. A survey of water production in 61 comets from SOHO/SWAN observations of hydrogen Lyman-alpha: twenty-one years 1996–2016. *Icarus* **317**, 610–620 (2019). 30270935 10.1016/j.icarus.2018.08.031 PMC6155483

[34] Hu, X., Gundlach, B., von Borstel, I., Blum, J. & Shi, X. Effect of radiative heat transfer in porous comet nuclei: case study of 67P/Churyumov–Gerasimenko. *Astron. Astrophys.* **630**, A5 (2019).

[35] Weissman, P. R., Bottke, W. F. Jr & Levison, H. F. in *Asteroids III* (eds Bottke, W. F. Jr et al.) 669–686 (Univ. Arizona Press, 2002).

[36] Meech, K. & Raymond, S. N. in *Planetary Astrobiology* (eds Meadows, V. S. et al.) 325–354 (Univ. Arizona Press, 2020).

[37] Marty, B. et al. Origins of volatile elements (H, C, N, noble gases) on Earth and Mars in light of recent results from the ROSETTA cometary mission. *Earth Planet. Sci. Lett.* **441**, 91–102 (2016).

[38] Hartogh, P. et al. Ocean-like water in the Jupiter-family comet 103P/Hartley 2. *Nature* **478**, 218–220 (2011). 21976024 10.1038/nature10519

[39] Altwegg, K. et al. 67P/Churyumov–Gerasimenko, a Jupiter family comet with a high D/H ratio. *Science* **347**, 1261952 (2015). 25501976 10.1126/science.1261952

[40] Mommert, M. et al. The discovery of cometary activity in near-Earth asteroid (3552) Don Quixote. *Astrophys. J.* **781**, 25 (2014).

[41] Kelley, M. S. P. et al. Spectroscopic identification of water emission from a main-belt comet. *Nature* **619**, 720–723 (2023). 37187210 10.1038/s41586-023-06152-y PMC10371862

[42] Kurlander, J. A. et al. Predictions of the LSST Solar System yield: near-Earth objects, main belt asteroids, Jupiter Trojans, and trans-Neptunian objects. *Astron. J.* **170**, 99 (2025).

[43] Daly, R. T. et al. Successful kinetic impact into an asteroid for planetary defence. *Nature* **616**, 443–447 (2023). 36858073 10.1038/s41586-023-05810-5 PMC10115643

[44] Mathias, D. L., Wheeler, L. F. & Dotson, J. L. A probabilistic asteroid impact risk model: assessment of sub-300 m impacts. *Icarus* **289**, 106–119 (2017).

[45] Flekkøy, E. G., Luu, J. & Toussaint, R. The interstellar object ’Oumuamua as a fractal dust aggregate. *Astrophys. J. Lett.* **885**, L41 (2019).

[46] Luu, J. X., Flekkøy, E. G. & Toussaint, R. ’Oumuamua as a cometary fractal aggregate: the “dust bunny” model. *Astrophys. J. Lett.* **900**, L22 (2020).

[47] Moro-Martín, A. Could 1I/’Oumuamua be an icy fractal aggregate? *Astrophys. J. Lett.* **872**, L32 (2019).

[48] Bergner, J. B. & Seligman, D. Z. Acceleration of 1I/‘Oumuamua from radiolytically produced H_2_ in H_2_O ice. *Nature* **615**, 610–613 (2023). 36949336 10.1038/s41586-022-05687-w

[49] Desch, S. J. & Jackson, A. P. 1I/‘Oumuamua as an N_2_ ice fragment of an exo Pluto surface II: generation of N_2_ ice fragments and the origin of ‘Oumuamua. *J. Geophys. Res. Planets* **126**, e06807 (2021).

[50] Seligman, D. & Laughlin, G. Evidence that 1I/2017 U1 (’Oumuamua) was composed of molecular hydrogen ice. *Astrophys. J. Lett.* **896**, L8 (2020).

[51] Desch, S. J. & Jackson, A. P. Some pertinent issues for interstellar panspermia raised after the discovery of 1I/‘Oumuamua. *Astrobiology* **22**, 1400–1413 (2022). 36475963 10.1089/ast.2021.0199

[52] Farnocchia, D., Chesley, S. R., Milani, A., Gronchi, G. F. & Chodas, P. W. in *Asteroids IV* (eds Michel, P. et al.) 815–834 (Univ. Arizona Press, 2015).

[53] Eggl, S., Farnocchia, D., Chamberlin, A. B. & Chesley, S. R. Star catalog position and proper motion corrections in asteroid astrometry II: the Gaia era. *Icarus* **339**, 113596 (2020).

[54] Vereš, P., Farnocchia, D., Chesley, S. R. & Chamberlin, A. B. Statistical analysis of astrometric errors for the most productive asteroid surveys. *Icarus* **296**, 139–149 (2017).

[55] Marsden, B. G., Sekanina, Z. & Yeomans, D. K. Comets and nongravitational forces. V. *Astron. J.* **78**, 211 (1973).

[56] Buzzoni, B. et al. The ESO Faint Object Spectrograph and Camera / EFOSC. *Messenger* **38**, 9 (1984).

[57] Boulade, O. et al. in *Instrument Design and Performance for Optical/Infrared Ground-based Telescopes* Society of Photo-Optical Instrumentation Engineers (SPIE) Conference Series Vol. 4841 (eds Iye, M. & Moorwood, A. F. M.) 72–81 (SPIE, 2003).

[58] Appenzeller, I. et al. Successful commissioning of FORS1—the first optical instrument on the VLT. *Messenger* **94**, 1–6 (1998).

[59] Bertin, E. & Arnouts, S. SExtractor: software for source extraction. *Astron. Astrophys. Suppl. Ser.* **117**, 393–404 (1996).

[60] Barbary, K. Sep: source extractor as a library. *J. Open Source Softw.* **1**, 58 (2016).

[61] Beroiz, M., Cabral, J. & Sanchez, B. Astroalign: a Python module for astronomical image registration. *Astron. Comput.* **32**, 100384 (2020).

[62] Giorgini, J. D. et al. JPL’s on-line Solar System data service. In *AAS/Division for Planetary Sciences Meeting #28* 25.04 (1996).

[63] Vavilov, D. E. & Carry, B. Rotation periods of asteroids from light curves of TESS data. *Astron. Astrophys.* **693**, A66 (2025).

[64] Finson, M. L. & Probstein, R. F. A theory of dust comets. I. Model and equations. *Astrophys. J.* **154**, 327–352 (1968).

[65] Rotundi, A. et al. Dust measurements in the coma of comet 67P/Churyumov–Gerasimenko inbound to the Sun. *Science* **347**, aaa3905 (2015). 25613898 10.1126/science.aaa3905

[66] Britt, D. T., Yeomans, D., Housen, K. & Consolmagno, G. in *Asteroids III* (eds Bottke, W. F. Jr et al.) 485–500 (Univ. Arizona Press, 2002).

[67] Moreno, F. et al. The dust environment of comet 67P/Churyumov–Gerasimenko from Rosetta OSIRIS and VLT observations in the 4.5 to 2.9 au heliocentric distance range inbound. *Astron. Astrophys.* **587**, A155 (2016).

[68] Meech, K. J. & Svoren, J. in *Comets II* (eds Festou, M. C. et al.) 317–335 (Univ. Arizona Press, 2004).

[69] Nolan, M. C., Harmon, J. K., Howell, E. S., Campbell, D. B. & Margot, J.-L. Detection of large grains in the coma of Comet C/2001 A2 (LINEAR) from Arecibo radar observations. *Icarus* **181**, 432–441 (2006).

[70] Fulle, M. et al. Evolution of the dust size distribution of comet 67P/Churyumov–Gerasimenko from 2.2 au to perihelion. *Astrophys. J.* **821**, 19 (2016).

[71] Jewitt, D. et al. Hubble Space Telescope investigation of main-belt comet 133P/Elst–Pizarro. *Astron. J.* **147**, 117 (2014).

[72] Ostro, S. J. et al. Radar observations of asteroid 1620 Geographos. *Icarus* **121**, 46–66 (1996).

[73] Magri, C. et al. Radar observations and a physical model of asteroid 1580 Betulia. *Icarus* **186**, 152–177 (2007).

[74] Benner, L. A. M. et al. Near-Earth asteroid surface roughness depends on compositional class. *Icarus* **198**, 294–304 (2008).

[75] Virkki, A. K. et al. Arecibo planetary radar observations of near-Earth asteroids: 2017 December–2019 December. *Planet. Sci. J.* **3**, 222 (2022).

[76] Virkki, A. & Muinonen, K. Radar scattering by planetary surfaces modeled with laboratory-characterized particles. *Icarus* **269**, 38–49 (2016).

[77] Hickson, D. C., Virkki, A. K., Perillat, P., Nolan, M. C. & Bhiravarasu, S. S. Polarimetric decomposition of near-Earth asteroids using Arecibo radar observations. *Planet. Sci. J.* **2**, 30 (2021).

[78] Harmon, J. K., Nolan, M. C., Ostro, S. J. & Campbell, D. B. in *Comets II* (eds Festou, M. C. et al.) 265 (Univ. Arizona Press, 2004).

[79] Harmon, J. K. & Nolan, M. C. Radar observations of Comet 2P/Encke during the 2003 apparition. *Icarus* **176**, 175–183 (2005).

[80] Harmon, J. K. et al. Radar observations of Comet P/2005 JQ5 (Catalina). *Icarus* **184**, 285–288 (2006).

[81] Harmon, J. K., Nolan, M. C., Giorgini, J. D. & Howell, E. S. Radar observations of 8P/Tuttle: a contact-binary comet. *Icarus* **207**, 499–502 (2010).

[82] Harmon, J. K., Nolan, M. C., Howell, E. S., Giorgini, J. D. & Taylor, P. A. Radar observations of comet 103P/Hartley 2. *Astrophys. J. Lett.* **734**, L2 (2011).

[83] Ostro, S. J., Campbell, D. B. & Shapiro, I. I. Mainbelt asteroids: dual-polarization radar observations. *Science* **229**, 442–446 (1985). 17738665 10.1126/science.229.4712.442

[84] Magri, C., Consolmagno, G. J., Ostro, S. J., Benner, L. A. M. & Beeney, B. R. Radar constraints on asteroid regolith compositions using 433 Eros as ground truth. *Meteorit. Planet. Sci.* **36**, 1697–1709 (2001).

